# Inferring synteny between genome assemblies: a systematic evaluation

**DOI:** 10.1186/s12859-018-2026-4

**Published:** 2018-01-30

**Authors:** Dang Liu, Martin Hunt, Isheng J Tsai

**Affiliations:** 1 Genome and Systems Biology Degree Program, National Taiwan University and Academia Sinica, Taipei, Taiwan; 2 Biodiversity Research Center, Academia Sinica, Taipei, Taiwan; 3 Nuffield Department of Clinical Medicine, Experimental Medicine Division, John Radcliffe Hospital, University of Oxford, Oxford, OX1 1NF UK; 4 European Bioinformatics Institute (EMBL-EBI), Wellcome Trust Genome Campus, Hinxton, Cambridge, CB10 1SD UK

**Keywords:** Genome synteny, Assembly quality, Comparative genomics, Nematode genomes

## Abstract

**Background:**

Genome assemblies across all domains of life are being produced routinely. Initial analysis of a new genome usually includes annotation and comparative genomics. Synteny provides a framework in which conservation of homologous genes and gene order is identified between genomes of different species. The availability of human and mouse genomes paved the way for algorithm development in large-scale synteny mapping, which eventually became an integral part of comparative genomics. Synteny analysis is regularly performed on assembled sequences that are fragmented, neglecting the fact that most methods were developed using complete genomes. It is unknown to what extent draft assemblies lead to errors in such analysis.

**Results:**

We fragmented genome assemblies of model nematodes to various extents and conducted synteny identification and downstream analysis. We first show that synteny between species can be underestimated up to 40% and find disagreements between popular tools that infer synteny blocks. This inconsistency and further demonstration of erroneous gene ontology enrichment tests raise questions about the robustness of previous synteny analysis when gold standard genome sequences remain limited. In addition, assembly scaffolding using a reference guided approach with a closely related species may result in chimeric scaffolds with inflated assembly metrics if a true evolutionary relationship was overlooked. Annotation quality, however, has minimal effect on synteny if the assembled genome is highly contiguous.

**Conclusions:**

Our results show that a minimum N50 of 1 Mb is required for robust downstream synteny analysis, which emphasizes the importance of gold standard genomes to the science community, and should be achieved given the current progress in sequencing technology.

**Electronic supplementary material:**

The online version of this article (10.1186/s12859-018-2026-4) contains supplementary material, which is available to authorized users.

## Background

The essence of comparative genomics lies in how we compare genomes to reveal species’ evolutionary relationships. Advances in sequencing technologies have enabled the exploration of many new genomes across all domains of life [1–8]. Unfortunately, in most instances correctly aligning even just two genomes at base-pair resolution can be challenging. A genome usually contains millions or billions of nucleotides and is different from the genome of a closely related species as a result of evolutionary processes such as sequence mutations, chromosomal rearrangements, and gene family expansion or loss. There are high computational costs when trying to align and assign multiple copies of DNA that are identical to each other, such as segmental duplications and transposable elements [9–12]. In addition, it has been shown that popular alignment methods disagree with each other [9].

An alternative and arguably more practical approach relies on the identification of synteny blocks [13, 14], first described as homologous genetic loci that co-occur on the same chromosome [15, 16]. Synteny blocks are more formally defined as regions of chromosomes between genomes that share a common order of homologous genes derived from a common ancestor [17, 18]. Alternative names such as conserved synteny or collinearity have been used interchangeably [13, 19–22]. Comparisons of genome synteny between and within species have provided an opportunity to study evolutionary processes that lead to diversity of chromosome number and structure in many lineages across the tree of life [23, 24]; early discoveries using such approaches include chromosomal conserved regions in nematodes and yeast [25–27], evolutionary history and phenotypic traits of extremely conserved Hox gene clusters across animals and MADS-box gene family in plants [28, 29], and karyotype evolution in mammals [30] and plants [31]. Analysis of synteny in closely related species is now the norm for every new published genome. However, assembly quality comes into question as it has been demonstrated to affect subsequent analysis such as annotation or rate of lateral transfer [32, 33].

In general, synteny identification is a filtering and organizing process of all local similarities between genome sequences into a coherent global picture [34]. The most intuitive way to identify synteny would be to establish from selective genome alignments [35, 36], but levels of nucleotide divergence between species may make such methodologies challenging. Instead, many tools use orthologous relationships between protein-coding genes as anchors to position statistically significant local alignments. Approaches include the use of a directed acyclic graph [37, 38], a gene homology matrix (GHM) [39], and an algorithm using reciprocal best hits (RBH) [40]. All of these methods generally agree on long synteny blocks, but have differences in handling local shuffles as well as in the resolution of synteny identification [34, 40]. Better resolution of micro-rearrangements in synteny patterns has been shown when using an improved draft genome of *Caenorhabditis briggsae* versus *Caenorhabditis elegans* [26, 41]. Hence, synteny analysis depends highly on assembly quality. For example, missing sequences in an assembly may lead to missing gene annotations and subsequently missing orthologous relationships [42]. With respect to assembly contiguation, it still remains unclear whether assembly fragmentation affects homology assignments for identifying anchors, sequence arrangements for examining order and gaps, or other factors in synteny analysis.

In this study, we focus on how assembly quality affects the identification of genome synteny. We investigate the correlation between error rate (%) in detecting synteny and the level of assembly contiguation using five popular software packages (DAGchainer [37], i-ADHoRe [39], MCScanX [38], SynChro [40], and Satsuma [36]) on four nematodes: *Caenorhabditis elegans, Caenorhabditis briggsae*, *Strongyloides ratti,* and *Strongyloides stercoralis*. We also carried out and explored the effects of three scenarios associated with synteny analysis: gene ontology (GO) enrichment, reference-guided assembly scaffolding, and annotation quality. Our findings show that assembly quality does matter in synteny analysis, and fragmented assemblies ultimately lead to erroneous findings. In addition, the true evolutionary relationship may be lost if a fragmented assembly is scaffolded using a reference-guided approach. Our main aim here is to determine a minimum contiguation of assembly for subsequent synteny analysis to be trustworthy, which should be possible using the latest sequencing technologies [43].

## Results

### Definition of synteny block, break and coverage

**Fig. 1:**
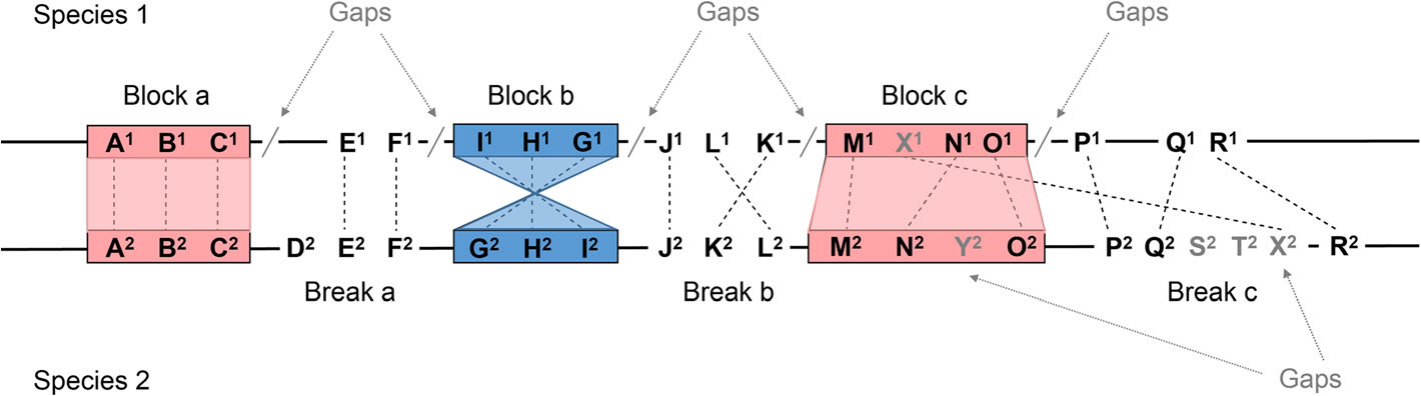
Definition of synteny block and break. Genes located on chromosomes of two species are denoted in letters. Each gene is associated with a number representing the species they belong to (species 1 or 2). Orthologous genes are connected by dashed lines and genes without an orthologous relationship are treated as gaps in synteny programs. Under the criterion of at least three orthologous genes (anchors): a synteny block can be orthologs with the same order (block a), with reversed order (block b), or allowing some gaps (block c). In contrast, cases of causing a synteny break can be lack of orthologs (break a), co-arranged gene order (break b) or gaps (break c)

We begin with some terminology used throughout this study. As shown in Fig. 1, a synteny block is defined as a region of genome sequence spanning a number of genes that are orthologous and co-arranged with another genome. Orientation is not considered (Fig. 1, block a and b). The minimum number of co-arranged orthologs said to be the anchors can be set and vary between different studies. A higher number of minimum anchors may result in fewer false positives, but also a more conservative estimate of synteny blocks (Additional file 1: Figure S1). In some programs, some degrees of gaps—defined as the number of skipped genes or the length of unaligned nucleotides—are tolerated (Fig. 1, block c). A score is usually calculated, and synteny breaks are regions that do not satisfy a certain score threshold. Possible scenarios that lead to synteny breaks include a lack of anchors in the first place (Fig. 1, break a), a break in anchor order (Fig. 1, break b), or gaps (Fig. 1, break c). Genome insertions and duplications may cause oversized gaps. An example is break c in Fig. 1, which is due to either a large unaligned region (Fig. 1, P^1^-Q^1^ and Q^2^-R^2^) or a high number of skipped genes (Fig. 1, S^2^-T^2^-X^2^ within Q^2^-R^2^). Alternatively, an inversion (Fig. 1, orthologs K and L), deletion, or transposition (Fig. 1, ortholog X) may cause a loss of anchors (Fig. 1, gene D in species 1) or a break in the arrangement of anchors. Typically, synteny coverage is commonly used as a summary metric obtained by taking the summed length of blocks and dividing it by genome size. Note that synteny coverage is asymmetrical between reference and query genomes, as demonstrated by the difference of block length in block c (Fig. 1).

### Evaluation of synteny identification programs in fragmented assemblies

**Fig. 2:**
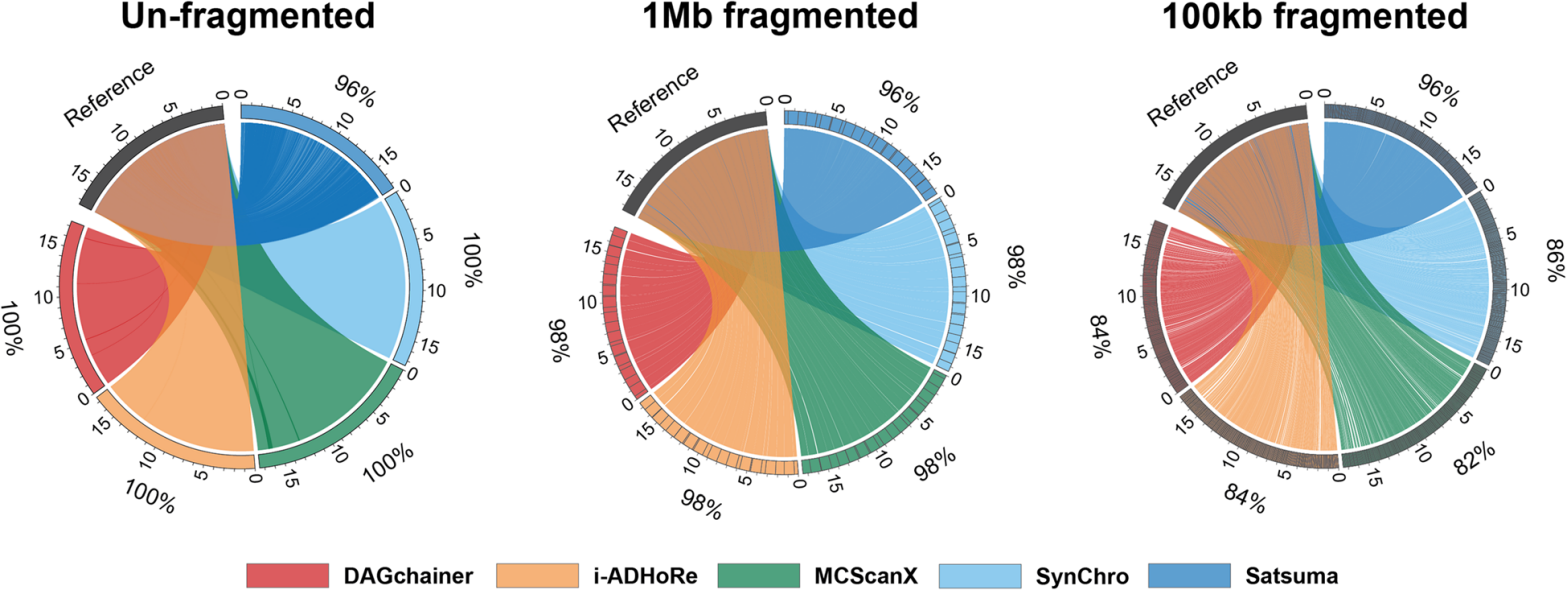
Synteny blocks identified between un-fragmented and fragmented *C. elegans* chromosome IV. The original sequence is used as the reference and coloured in black. Established synteny regions (outer number stands for synteny coverage) of the 5 different program packages: DAGchainer (red), i-ADHoRe (yellow), MCScanX (green), SynChro (light blue), and Satsuma (blue) are joined to query sequences with different levels of fragmentation (un-fragmented, 1 Mb and 100 kb fragmented). Chromosome positions are labeled in megabases (Mb). For plots of other chromosomes see Additional file 3: Figure S2 and Additional file 4: Figure S3

There are several programs developed to identify synteny blocks, which can produce quite different results [14]. Our first aim is to systematically assess the synteny identification of four popular anchor-based tools: DAGchainer [37], i-ADHoRe [39], MCScanX [38], SynChro [40] and one based solely on nucleotide alignments: Satsuma [36]. As whole genome alignments between bacteria, which have relatively small genomes, is becoming common practice [44], we conduct this study on species with larger genome sizes. We chose *Caenorhabditis elegans*, a model eukaryote with a 100 megabase (Mb) reference genome. Our first question was if these programs would produce 100% synteny coverage if the *C. elegans* genome was compared to itself. As expected, all anchor-based tools accurately achieved almost 100% synteny coverage, with the exception of Satsuma reaching 96% (Fig. 2).

We then fragmented the *C. elegans* genome into fixed intervals of either 100 kb, 200 kb, 500 kb or 1 Mb to evaluate the performance of different programs when using self-comparisons (see Methods). Synteny coverages of the fragmented assembly (query) against the original assembly (reference) were calculated for both query and reference sequences. Generally, synteny coverage decreased as the assembly was broken into smaller pieces. For example, an average of 16% decrease in synteny coverage was obtained using the assembly with fixed fragment size of 100 kb (Additional file 2: Table S1). Sites of fragmentation are highly correlated with synteny breaks in anchor-based programs (Fig. 2, Additional file 3: Figure S2, and Additional file 4: Figure S3). One explanation is that the fragmented assembly introduced breaks within genes that resulted in loss of anchors (Fig.1, break a), which can be common in real assemblies if introns contain hard to assemble sequences [32]. Another explanation is that the breaks between genes lead to the number of anchors not reaching the required minimum (Fig. 1, Break a). For the case of Satsuma, synteny identification was not affected by assembly fragmentation (Fig. 2, Additional file 3: Figure S2, and Additional file 4: Figure S3; Additional file 2: Table S1).

**Fig. 3:**
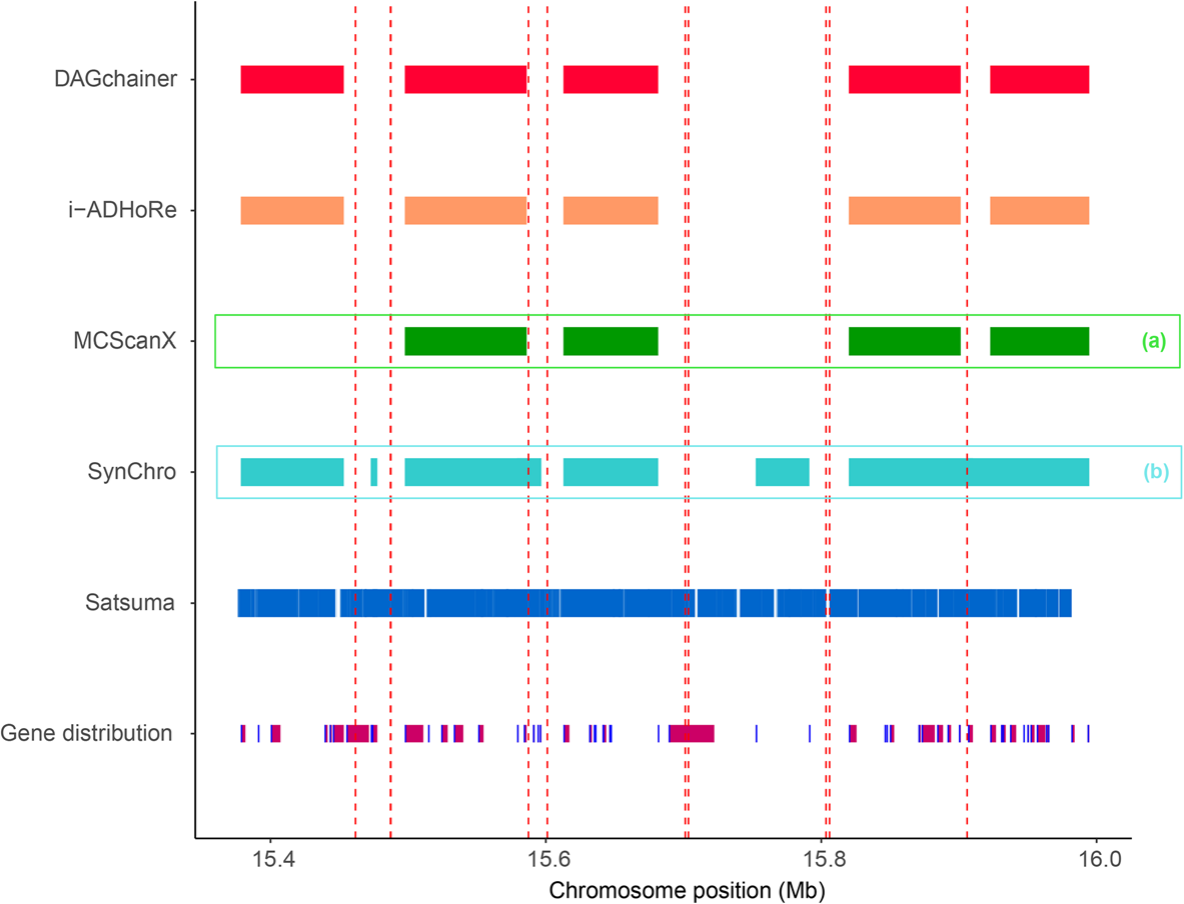
A zoomed-in 600 kb region of synteny identified between the reference *C. elegans* genome and a 100 kb fragmented assembly. Synteny blocks in fragmented assembly defined by the five detection programs DAGchainer (red), i-ADHoRe (yellow), MCScanX (green), SynChro (light blue), and Satsuma (blue) are drawn as rectangles. Fragmented sites are labeled by vertical red dashed lines. Genes are shown as burgundy rectangles, with gene starts marked using dark blue lines. Two scenarios are marked: a) a synteny block was not identified by MCScanX, and b) several synteny blocks only detected by SynChro

More fragmented assemblies led to greater differences in synteny coverage predicted between the four anchor-based tools (Fig. 2, Additional file 3: Figure S2, and Additional file 4: Figure S3). We carefully examined regions where synteny was predicted in some programs but not the other (Figs. 2 and 3). Figure 3 demonstrates such a case of disagreement. It is apparent that Satsuma is neither affected by genome fragmentation nor gene distribution (Fig. 3). For the other programs, DAGchainer and i-ADHoRe produced the same results, whilst MCScanX and SynChro detected less and more synteny, respectively (Fig. 3). MCScanX’s gap scoring scheme used a stricter synteny definition, and more synteny blocks can be identified when the gap threshold was lowered (Fig. 3, situation a; also see Additional file 5: Figure S4). SynChro has its own dedicated orthology assignment approach that assigns more homologous anchors (Fig. 3, situation b). Additionally, SynChro uses only 2 genes as anchors to form a synteny block, while the default is at least five gene anchors in other three tools (Fig. 3, situation b). Together, these results suggest that the default parameters set by different tools will lead to differences in synteny identification and need to be tuned before undertaking subsequent analysis.

### Contribution of assembly contiguation and intrinsic species effect to synteny analysis

**Fig. 4:**
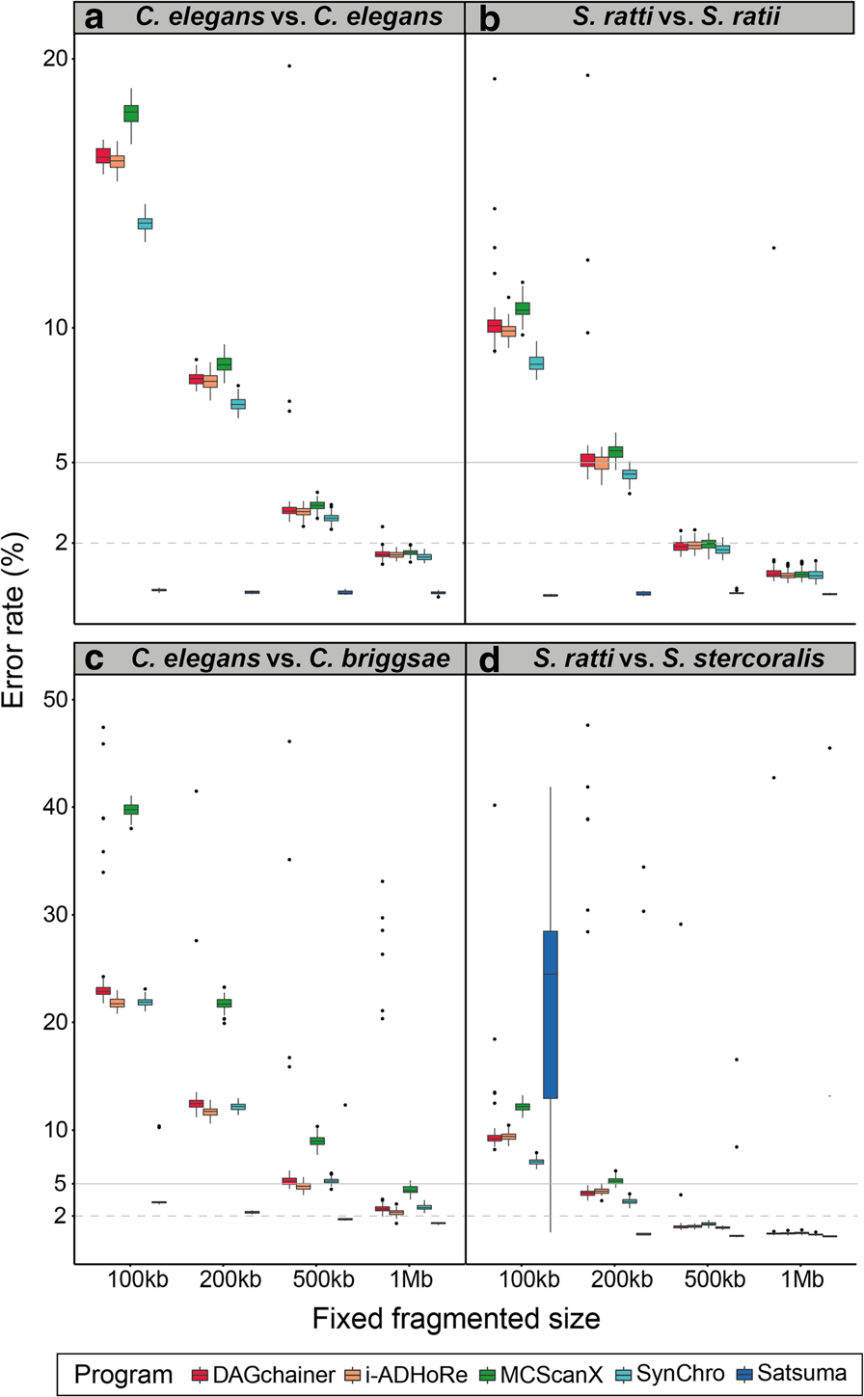
Error rate (%) of synteny identification in fragmented assemblies. The error rate is defined as the difference between the synteny coverage calculated with the original genome (almost 100%) and that in fragmented assemblies, where the original assembly was used as the reference in both cases. 5% and 2% error rate positions are marked by grey solid and dashed lines, respectively. Different pairs in synteny identification are separated in different panels. The upper panels are self-comparisons, while the bottom are comparisons between closely related species. Note that for a clear visualization of pattern changes, the scales of error rate are different between upper and bottom panels. Colors represent different types of synteny detection programs. The letters **a**, **b**, **c** and **d** denote the comparisons of *C. elegans* vs. *C. elegans*, *S. ratti* vs. *S. ratti*, *C. elegans* vs. *C. briggsae,* and *S. ratti* vs. *S. stercoralis* respectively

To quantify the effect of assembly contiguation in synteny analysis, we used four nematode genomes: *Caenorhabditis elegans, Caenorhabditis briggsae*, *Strongyloides ratti,* and *Strongyloides stercoralis*. Nematodes are useful models in synteny analysis as 1) extensive chromosomal rearrangement is a hallmark of their genome evolution [7, 25, 26, 45, 46] and 2) their genome sequences are highly contiguous and assembled into chromosomes [7, 25, 26, 45]. These two genera were also chosen to investigate the intrinsic species effect as they differ in gene density (Table 1). Our fragmentation approach was first used to break the *C. elegans* and *S. ratti* genomes into fixed sequence sizes of either 100 kb, 200 kb, 500 kb, or 1 Mb. Here, we define the error rate as the difference between the original synteny coverage (almost at 100%) and fragmented assembly. For each fixed length, the fragmentation was repeated 100 times for most programs so that assemblies got broken at different places to obtain a distribution; the fragmentation was only repeated 10 times in Satsuma due to its long run time. There is a positive correlation between error rate and level of fragmentation, except for synteny blocks detected by Satsuma (Fig. 4a and b; Additional file 2: Table S1). Amongst the four anchor-based tools, the median error rate can be as high as 18% for 100 kb fragmented assemblies (Additional file 2: Table S1) and the fragmentation has the largest effect in MCScanX and smallest in SynChro (Fig. 4a and b; Additional file 2: Table S1).

**Table 1:**
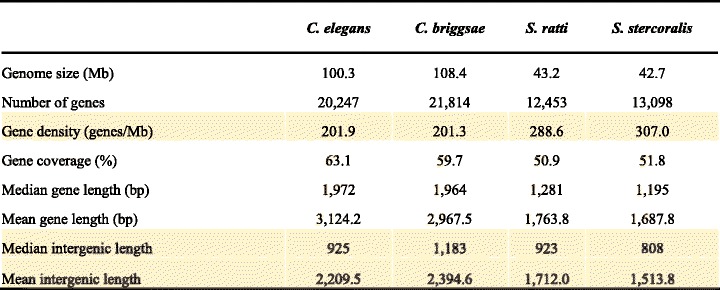
Genomic features of *Caenorhabditis* and *Strongyloides* species

Features that may play a key role in synteny detection are highlighted in yellow

A common analysis when reporting a new genome is inferring synteny against a closely related species. Hence, we reanalyzed synteny between *C. elegans* and *C. briggsae*, *S. ratti* and *S. stercoralis.* Satsuma found only 19% and 54% synteny in *C. elegans*-*C. briggsae* and *S. ratti*-*S. stercoralis* comparisons, respectively, presumably because of difficulty in establishing orthology at the nucleotide level (Additional file 2: Table S1). On average, the four anchor-based tools found 77% and 83% synteny between *C. elegans*-*C. briggsae* and *S. ratti*-*S. stercoralis* respectively (Additional file 2: Table S1). In contrast to the general agreement on within-species self-comparisons, the anchor-based tools varied considerably on these inter-species (i.e. more diverged) comparisons (Additional file 6: Figure S5 and Additional file 2: Table S1). For example, in the *C. elegans*-*C. briggsae* comparisons, a difference of 25% in synteny coverage was found between the results of i-ADHoRe and SynChro (Additional file 6: Figure S5 and Additional file 2: Table S1), while this tool variation was interestingly much lower in *S. ratti*-*S. stercoralis*—only a 9% difference (Additional file 2: Table S1). To increase the complexity, we fragmented *C. briggsae* and *S. stercoralis* into fixed sequence sizes using the same approach as previously mentioned and compared them with the genome of *C. elegans* and *S. ratti*, respectively. We found that MCScanX still underestimated synteny the most as the scaffold size decreased from 1 Mb to 100 kb. Strikingly, the median error rate was as high as 40% in *C. elegans-C. briggsae* but only 12% in *S. ratti*-*S. stercoralis* comparisons (Fig. 4c and d). The error rate is also as high as 40% and largely variable in the comparison between *S. ratti* and 100 kb fragmented *S. stercoralis* using Satsuma (Fig. 4d). This observation suggests that higher gene density leads to a more robust synteny detection in fragmented assemblies when more anchors (genes) are available in a given sequence (Additional file 1: Table 1 and Additional file 1: Figure S1).

### Synteny identification in real-world scenarios

**Fig. 5:**
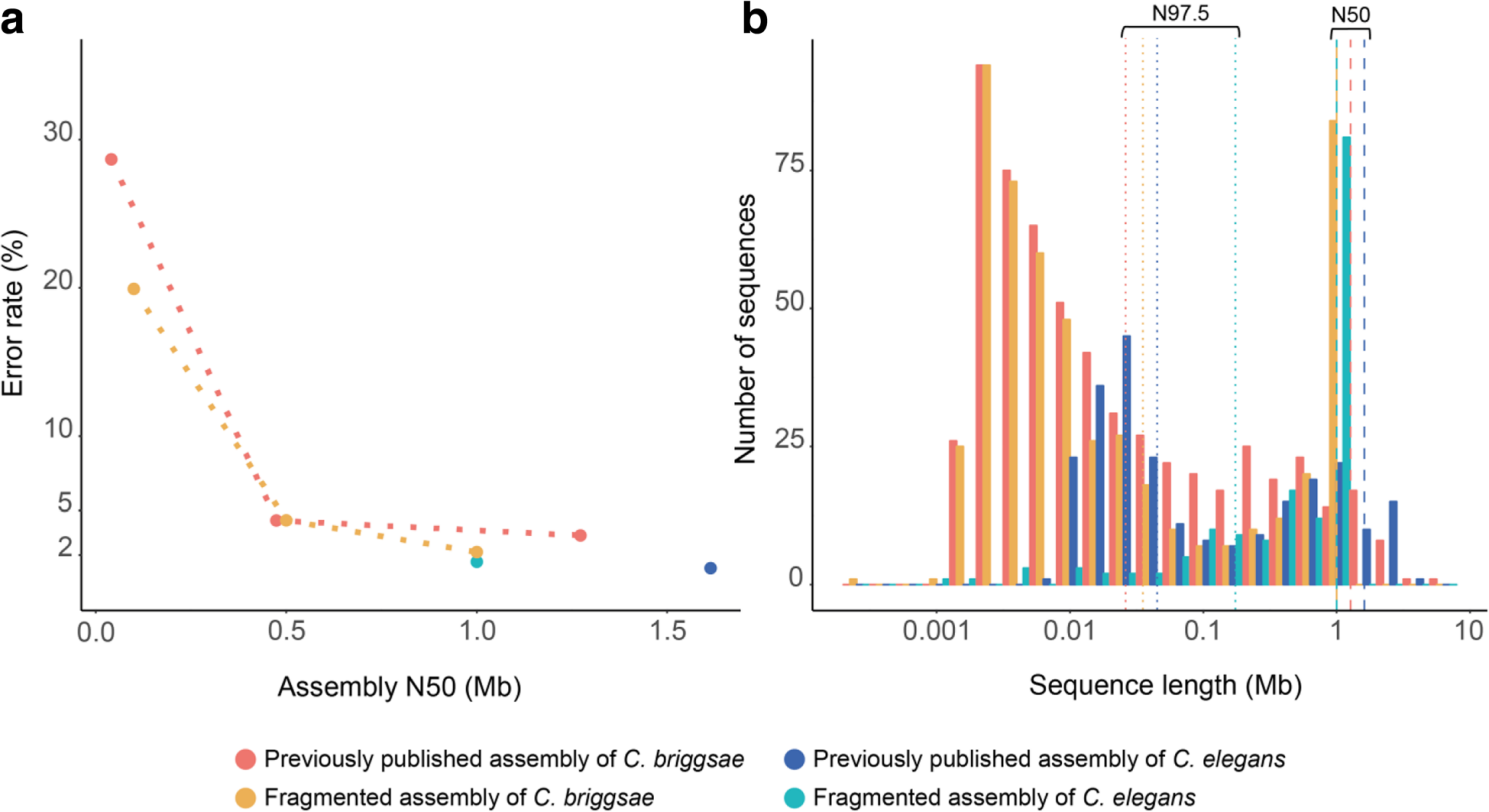
Relationship between error rate (%) in synteny identification and distribution of sequence length in assemblies. Different colors denote multiple sources of assembly. Panel **a** shows error rates (%) in synteny identification when assemblies compared against the *C. elegans* reference genome. Panel **b** demonstrates distributions of sequence length of assemblies with an N50 of around 1 Mb. Dashed and dotted lines specify the N50 and N97.5 respectively

To assess the robustness of our observations from the fragmentation approach, we sought to compare real assemblies of various contiguities. A recent publicly available genome of *C. elegans* using long reads data and three older versions of *C. briggsae* genomes assemblies were retrieved (see Methods). An error rate of 1.1% in synteny identified from DAGchainer was obtained when comparing the recent *C. elegans* assembly with N50 of 1.6 Mb against the reference, which is very similar to our 1 Mb fragmented assemblies of 1.5% (Fig. 5a). When we compared *C. elegans* against *C. briggsae* assemblies of different contiguation, error rates were negatively correlated with N50, regardless of how the *C. briggsae* assemblies were derived, i.e., simulated or published assemblies (Fig. 5a). The distribution of sequence length in the assemblies indicate that our fragmented approach of fixed sizes may not capture the sequence length residing at either tail of the distribution (Fig. 5b). The short sequences were abundant in published assemblies, but occupy less than 2.5% of the assemblies (as specified to the left of N97.5 in Fig. 5b). Nevertheless, in terms of synteny coverage, these results suggest that our fragmentation approach is robust.

### Erroneous findings from fragmented assemblies in synteny analysis

**Fig. 6:**
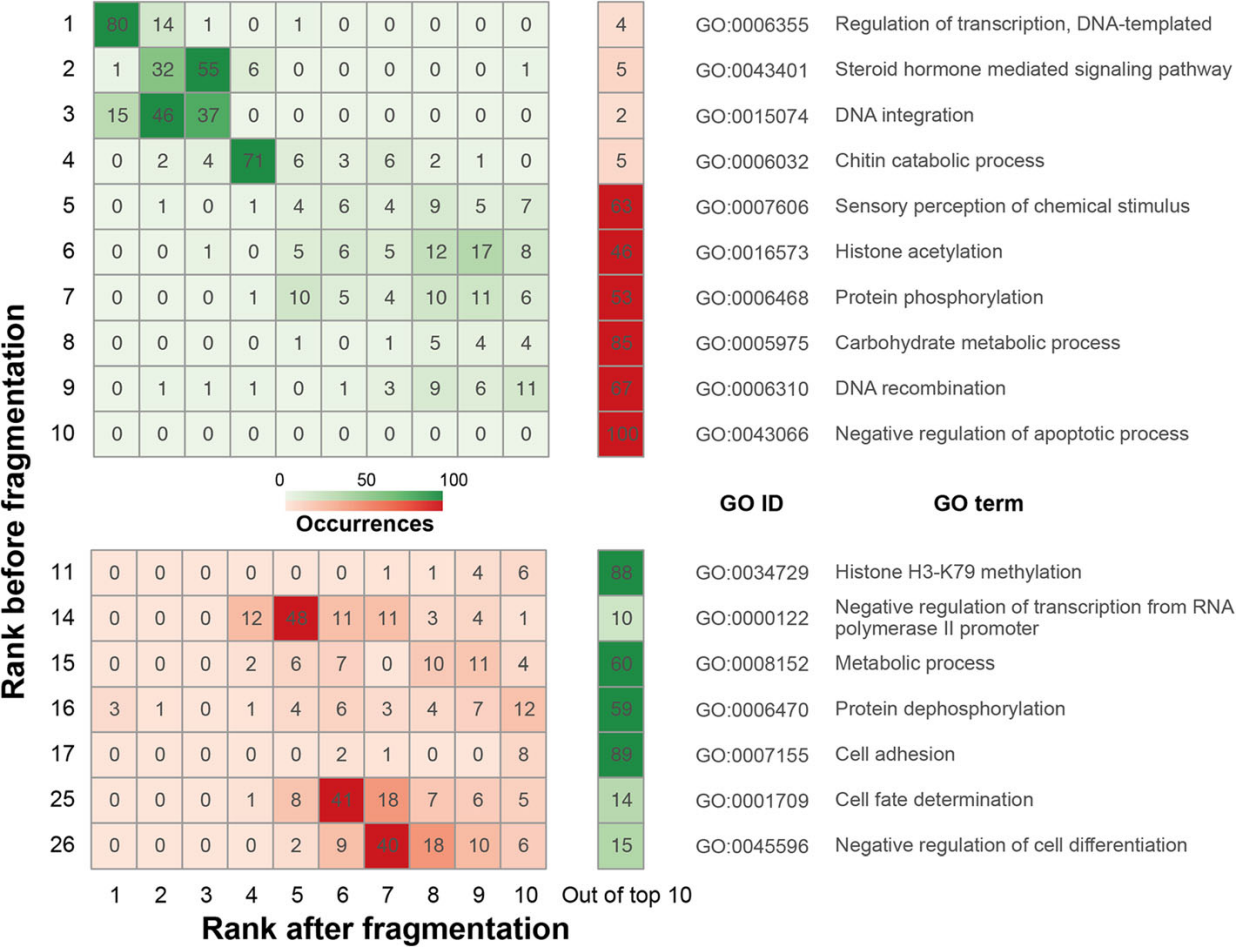
Comparison of gene ontology (GO) enriched terms in *C. briggsae* synteny breaks between *C. elegans* vs. *C. briggsae* and 100 replicates of *C. elegans* vs. 100 kb fragmented *C. briggsae*. Top ranks of GO terms in the original comparison are shown in the Y axis. For original top ranking GO terms, only those that appeared more than 10 times in top 10 ranks of after-fragmentation comparison replicates were displayed (see Additional file 7: Table S2 for more details). The X axis shows top 10 ranks and rank “out of top 10” in the comparison when assemblies were fragmented. The darkness of color is proportional to the occurrence of the GO term in that rank within 100 replicates. Regions in red are indications of occurred ranking errors. All GO categories have adjusted *p*-value < 0.01

Functional enrichment of genes of interest is often performed after the establishment of orthology and synteny [26, 47–50]. Synteny breaks are caused by rearrangements, the insertion of novel genes, or the presence of genes that are too diverged to establish an orthologous relationship or have undergone expansion or loss. Functions of these genes are often of interest in comparative genomics analyses. To further estimate the effect of poor assembly contiguation on synteny analysis, GO enrichment was performed on genes present in *C. briggsae* synteny breaks identified from DAGchainer in *C. elegans* vs. 100 kb fragmented *C. briggsae*. This approach was then repeated 100 times with assemblies fragmented randomly. We found that fragmented assemblies make GO terms that were originally not found in the top 100 ranks consistently appear in the top 10 during the 100 replicates (Fig. 6 and Additional file 7: Table S2). Furthermore, the orders of the original top 10 GO terms shifted in fragmented assemblies (Fig. 6 and Additional file 7: Table S2). In addition, the 10th top GO term failed to appear in the top 10 in 100 replicates (Fig. 6 and Additional file 7: Table S2). These results suggest that an underestimation of synteny relationship due to poor assembly contiguation can lead to a number of erroneous findings in subsequent analysis.

### True synteny may be compromised by reference-guided assembly methods

**Fig. 7:**
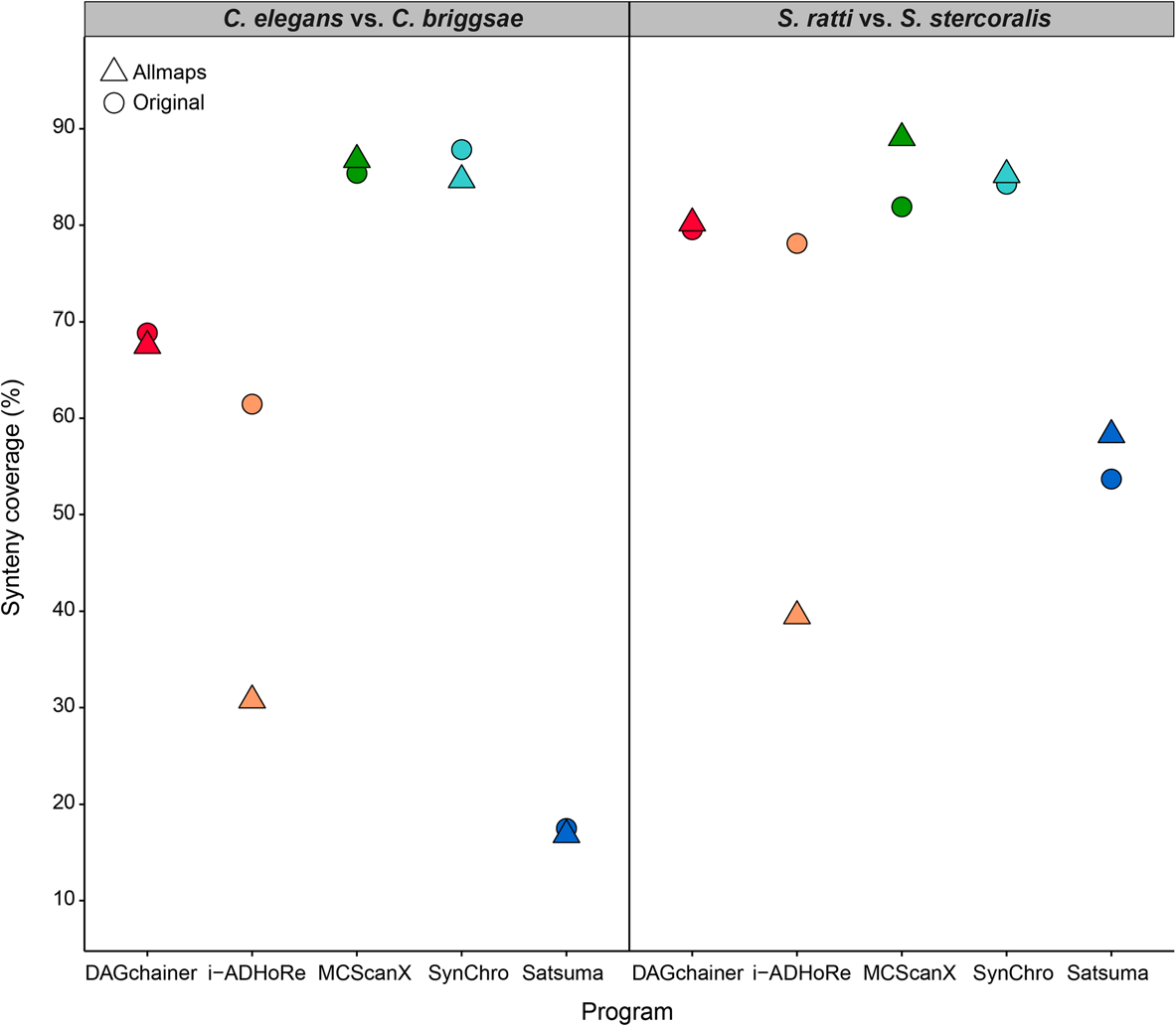
Synteny coverage (%) between *C. elegans* and *S. ratti* assemblies against original or ALLMAPS scaffolded assemblies from 100 kb fragmented assemblies of *C. briggsae* and *S. stercoralis*

**Fig. 8:**
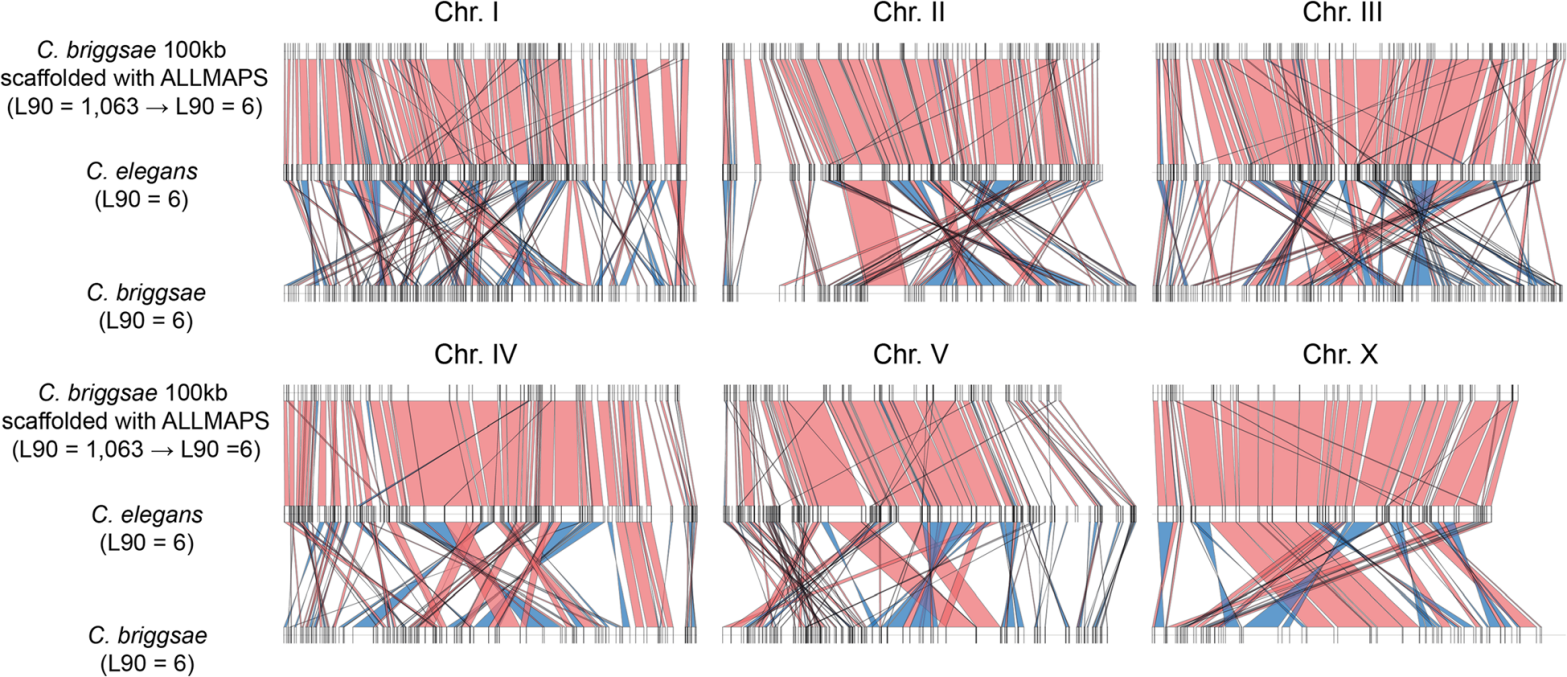
Synteny linkage of *C. elegans* vs. original *C. briggsae* assembly and *C. elegans* vs. ALLMAPS *C. briggsae* assembly. ALLMAPS assembly with L90 = 1063 from 100 kb fragmented *C. briggsae* assembly with L90 = 6 (top), original *C. elegans* assembly with L90 = 6 (middle) and original *C. briggsae* assembly with L90 = 6 (bottom) are shown in different horizontal lines. Vertical lines on chromosome lines show the start/end positions of the first/last gene in a synteny block. Each panel shows a separate chromosome. Block linkages in the same orientation are labeled in red, while those in inverted orientation are labeled in blue

Although assembly quality plays an important role in synteny analysis, it has been demonstrated that poor assembly contiguity of one species can be scaffolded by establishing synteny with a more contiguous assembly of a closely related species [42, 51–53]. However, we hypothesized that the true synteny relationship between two species may be incorrectly inferred when an assembly of one species is scaffolded based on another closely related species, by assuming the two genomes are syntenic. To investigate this, ALLMAPS [53] was used to order and orient sequences of 100 kb fragmented *C. briggsae* based on *C. elegans* as well as 100 kb fragmented *S. stercoralis* assembly based on *S. ratti*. ALLMAPS reduced the number of sequences in both fragmented assemblies impressively, increasing the N50 from 100 kb to 19 Mb and 15 Mb in *C. briggsae* and *S. stercoralis,* respectively (Additional file 8: Table S3). Synteny coverage from these scaffolded assemblies was even higher than the original fragmented 100 kb sequences in MCScanX, much lower in i-ADHoRe, and similar in DAGchainer, SynChro, and Satsuma (Fig. 7). However, despite synteny coverage being close to that of the original assemblies in DAGchainer and SynChro, further investigation of synteny block linkages in *C. elegans*-*C. briggsae* using identification from DAGchainer revealed frequent false ordering and joining of contigs, resulting in false synteny blocks. Intra-chromosomal rearrangements are common between *C. elegans* and *C. briggsae,* but the scaffolded assemblies produced by ALLMAPS show a false largely collinear relationship in the chromosomes between the two species (Fig. 8). Hence, if a true evolutionary relationship was not known, simply undergoing reference guided scaffolding would produce pseudo-high quality assemblies that may have ordering bias towards the reference genome and result in an incorrect assembly with inflated metrics.

### Annotation quality has little effect on synteny analysis

Genome annotation is a bridging step between genome assembly and synteny analysis. An incomplete annotation may lead to lack of homology information in synteny analysis. We compared synteny coverage in three datasets of *C. elegans* that differ in quality of annotation: 1) manually curated WormBase [24] *C. elegans* annotation, 2) optimized Augustus [54] annotation with its built-in *Caenorhabditis* species training set, and 3) semi-automated Augustus annotation with the BUSCO [55] nematoda species training set. In all cases, we found that synteny coverage varies at most 0.02% in the reference genome (Table 2). As a result, with a well-assembled genome and proper species training set, the quality of annotation has little effect on synteny analysis compared to assembly quality.

**Table 2:**
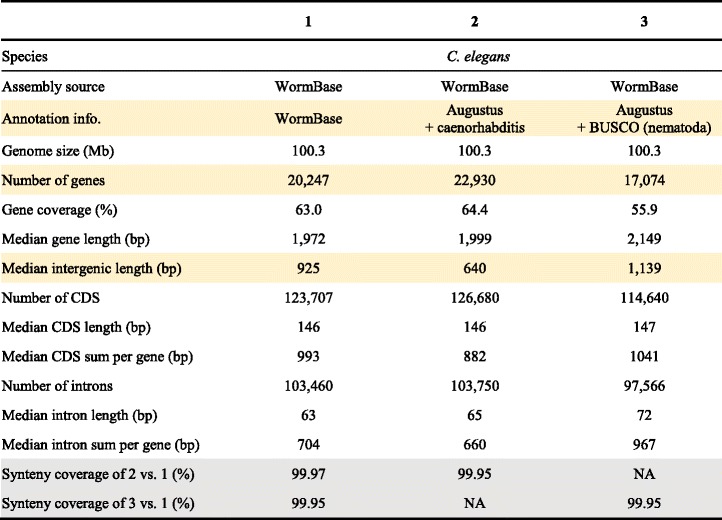
Statistics of *C. elegans* annotations used for synteny analysis

The statistics that relate to variation in annotation that may play a key role in synteny detection are highlighted in yellow. The result of synteny detection by DAGchainer is highlighted in grey

## Discussion

Synteny analysis is a practical way to investigate the evolution of genome structure [28–31, 56]. In this study, we have revealed how genome assembly contiguity affects synteny analysis. We present a simple scenario of breaking an assembly into a more fragmented state, which only mimics part of the poor assembly problem. Our genome fragmentation method randomly breaks sequences into same-sized pieces, which gives rise to a distribution of sequence length with peaks enriched in tiny sequences and fixed-size fragments (Fig. 5b). Real assemblies, which usually comprise a few large sequences and many more tiny sequences that are difficult to assemble because of their repetitive nature [25, 26], possess very different sequence length distributions (Fig. 5b). It is probable that we overestimated error rate in regions that can be easily assembled and underestimated error rate in regions that will be more fragmented, but overall synteny coverage were comparable (Fig. 5a). Note that some of the sequences in real assemblies may contain gaps (scaffolds) that will result in more missing genes and will result in further underestimation of synteny. Our results are quite similar when a de novo Pacbio *C. elegans* assembly and three older versions *C. briggsae* assemblies were compared to the reference *C. elegans* genome (Fig. 5a). The use of long read technology and advanced genome mapping such as the Hi-C approach [57] are becoming the norm for de novo assembly projects. Assemblies with lower contiguation were not compared here as we emphasize the responsibility of research groups to produce assemblies that are of the higher contiguity, made possible by long reads [58].

Synteny identification from different programs (i.e.*,* DAGchainer [37], i-ADHoRe [39], MCScanX [38], SynChro [40], and Satsuma [36]) performed across different species (*C. elegans*, *C. briggsae*, *S. ratti,* and *S. stercoralis*) have allowed us to examine the wide-ranging effects of assembly contiguation on downstream synteny analysis. Satsuma demonstrates fewer contiguation-dependent patterns as its detection of synteny relies on nucleotide alignments (Fig. 2). However, we show that Satsuma was only robust when comparing species with very low divergence, for example, between strains or assembly versions from the same species. Only ~ 19% of *C. elegans* and *C. briggsae* were identified as syntenic using Satsuma, and ~ 54% in *S. ratti*-*S. stercoralis* (Additional file 2: Table S1). Because initial identification of synteny coverage was low, any regions that failed to align in fragmented assemblies would consist of larger proportion of the original synteny coverage and lead to a higher error rate (Fig. 4d).

The other four programs, which are anchor-based, tend to produce the same results when the original assembly is compared to itself, but differ extensively when assemblies become fragmented (Fig. 2). It is interesting to note that DAGchainer and MCScanX use the same scoring algorithm for determining synteny regions, except that DAGchainer uses the number of genes without orthology assignment as gaps while MCScanX uses unaligned nucleotides. When comparing closely related species, results from the four anchor-based programs fluctuate even without fragmentation in *Caenorhabditis* species, while the pattern remains similar to self-comparison in *Strongyloides* species (Fig. 4). Sensitivity in synteny identification drops sharply as the genome assembly becomes fragmented, and thus genome assembly contiguation must be considered when inferring synteny relationships between species. Our fragmentation approach only affects N50, which mostly leads to loss of anchors in synteny analysis. Other scenarios such as unknown sequences (NNNs) in the assembly, rearrangements causing a break in anchor ordering (Fig. 1, break b), or insertions/deletions (Fig. 1, break c) were not addressed and may lead to greater inaccuracies.

We have shown that genomic features such as gene density and length of intergenic regions play an essential role during the process of synteny identification (Fig. 4; Tables 1 and Additional file 2: Table S1). Synteny identification can be established more readily in species with higher gene density or shorter intergenic space, which is related to the initial setting of minimum anchors needed for synteny identification (Fig. 1 and Additional file 1: Figure S1). Repetitiveness of paralogs is another factor in how anchors were chosen from homology assignments. For example, we found that synteny coverage is low along chromosomal arm regions of *C. elegans* in both self-comparison and versus *C. briggsae*, which has been reported to have expansion of G protein-coupled receptor gene families [25] (Fig. 2 and Additional file 6: Figure S5). Nevertheless, this case may be a result of a combination of repetitive paralogs and high gene density.

Interestingly, synteny comparison with scaffolded assemblies using ALLMAPS [53] exhibited unexpected variation among programs. Unfortunately, we did not resolve the reason behind the sharp decrease in synteny coverage from i-ADHoRe (Fig. 7). Nevertheless, we have shown that it is dangerous to scaffold an assembly using a reference from closely related species without a priori information about their synteny relationship. Subsequent synteny identification would be misleading if the same reference was compared again [59] (Fig. 8). We also considered the interplay between genome annotation, assembly and synteny identification. Although it may be intuitive to assume lower annotation quality can lead to errors in synteny analysis, we demonstrated that such influence was minimal if an initial genome assembly of good contiguation is available (Table 2).

## Conclusions

In conclusion, this study has demonstrated that a minimum quality of genome assembly is essential for synteny analysis. To keep the error rate below 5% in synteny identification, we suggest that an N50 of 200 kb and 1 Mb is required when gene density of species of interest are 290 and 200 genes per Mb, respectively (Tables 1 and Additional file 1: Figure S1). This is a minimum standard, and a higher N50 may be required for other species with lower gene density or highly expanded gene families.

## Methods

### Data preparation and fragmentation simulation

**Fig. 9:**
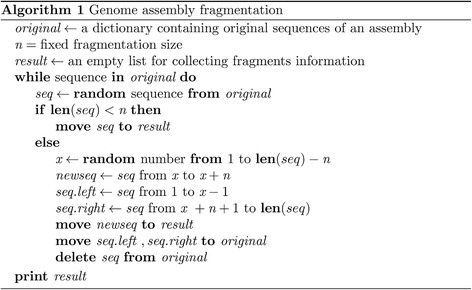
Pseudocode of genome assembly fragmentation

Assemblies and annotations of *C. elegans* and *C. briggsae* (release WS255), *S. ratti* and *S. stercoralis* (release WBPS8) were obtained from WormBase (http://www.wormbase.org/) [24]. A new assembly of *C. elegans* using long reads was obtained from a Pacific Bioscienceces dataset (https://github.com/PacificBiosciences/DevNet/wiki/C.-elegans-data-set). Initially published assemblies of *C. briggsae* were obtained from UCSC Genome Browser (http://hgdownload.cse.ucsc.edu/downloads.html#c_briggsae). The N50 of long reads assembled *C. elegans* genome, cb1 final version of *C. briggsae* genome, cb1 supercontig version of *C. briggsae* genome and cb1 contig version of *C. briggsae* genome are ~ 1.6 Mb, ~ 1.3 Mb, 474 kb and 41 kb respectively. Gene models of these assemblies were annotated de novo using Augustus [54]. Since some genes produce multiple alternative splicing isoforms and all of these isoforms represent one gene (locus), we used the longest isoform as a representative. Further, non-coding genes were also filtered out from our analysis. To simulate the fragmented state of assemblies, a script was made to randomly break scaffolds into fixed size fragments (Pseudocode shown in Fig. 9; scripts available at https://github.com/dangliu/Assembly-breaking.git). Sequences shorter than the fixed length were kept unchanged.

### Identification of Synteny blocks

The four anchor-based programs DAGchainer [37], i-ADHoRe [39] (v3.0), MCScanX [38] and SynChro [40], and the nucleotide alignment-based Satsuma [36], were used to identify synteny blocks. Settings for each program were modified to resemble each other on the results of *C. elegans* vs. *C. elegans*, where synteny should be close to 100%, with the exception of default setting in Satsuma. All of the anchor-based programs use gene orthology to find anchor points during process of synteny blocks detection. For DAGchainer, i-ADHoRe and MCScanX, we obtained gene orthology from OrthoFinder [60] (v0.2.8). SynChro has an implemented program called OPSCAN to scan for gene orthology. We arranged the following parameters for each program: DAGchainer (accessory script filter_repetitive_matches.pl was run with option 5 before synteny identification as recommended by manual; options: -Z 12 -D 10 -A 5 -g 1), i-ADHoRe (only top 1 hit of each gene in input blast file was used as recommended; options: cluster_type = collinear, alignment_method = gg2, max_gaps_in_alignment = 10, tandem_gap = 5, gap_size = 10, cluster_gap = 10, q_value = 0.9, prob_cutoff = 0.001, anchor_points = 5, level_2_only = false), MCScanX (only top 5 hits of each gene in the input blast file was used as suggested; options: default) and SynChro (options: 0 6; 0 for all pairwise, and 6 for delta of RBH genes). To calculate synteny coverage, the total length of merged synteny blocks along scaffolds was divided by total assembly size.

### Data analysis

Visualization of synteny linkages was made by R (v3.3.1) and circos [61] (v0.69–4). Gene ontology enrichment analysis was performed using the topGO [62] (v1.0) package in R and only focused on Biological Process (options: nodeSize = 3, algorithm = “weight01”, statistic = “Fisher”). Gene ontology associations files for *C. elegans* and *C. briggsae* were downloaded from WormBase WS255 [24]. Gene orthology was assigned by OrthoFinder [60]. Then, assemblies were scaffolded using ALLMAPS [53] with a reference guided approach. De novo annotations of *C. elegans* were predicted using either the manually trained species parameter or from BUSCO [55] (v2.0).

## Additional file

Additional file 1: Figure S1. Synteny coverage for different numbers of minimum anchors using DAGchainer. The Y axis shows synteny coverage (%). The X axis is the number of minimum anchors needed to identify a synteny block from 2 to 8. The 4 colorsare 4 combinations of synteny detection among species: *C. elegans* vs. *C. elegans* (CEvsCE, green), *C. elegans* vs. *C. briggsae* (CEvsCBG, orange), *S. ratti* vs. *S. ratti* (SRvsSR, blue) and *S. ratti* vs. *S. stercoralis* (SRvsSS, purple). (PNG 112 kb)
Additional file 2: Table S1. Quantification of synteny coverage and error rate. (DOCX 1 kb)
Additional file 3: Figure S2. Synteny blocks in *C. elegans* vs. 1Mb fragmented *C. elegans*. Chromosomes are separated into panels labelled with Roman numerals. The Y axis stands for categories of distribution. Synteny blocks defined by five detection programs: DAGchainer (red), i-ADHoRe (yellow), MCScanX (green), SynChro (light blue), and Satsuma (blue) are drawn as rectangles. Gene distribution is represented by the bottom smaller rectangles in burgundy. The X axis is the chromosome position. (PNG 286 kb)
Additional file 4: Figure S3. Synteny blocks in *C. elegans* vs. 100kb fragmented *C. elegans*. Chromosomes are separated into panels labelled with Roman numerals. The Y axis stands for categories of distribution. Synteny blocks defined by five detection programs: DAGchainer (red), i-ADHoRe (yellow), MCScanX (green), SynChro (light blue), and Satsuma (blue) are drawn as rectangles. Gene distribution is represented by the bottom smaller rectangles in burgundy. The X axis is the chromosome position. (PNG 322 kb)
Additional file 5: Figure S4. A zoomed-in 600kb region of synteny identified with lower gap threshold in MCScanX between the reference *C. elegans* genome and a 100kb fragmented assembly. The Y axis stands for categories of distribution. Synteny blocks in fragmented assembly defined by five detection programs: DAGchainer (red), i-ADHoRe (yellow), MCScanX (green), SynChro (light blue), and Satsuma (blue) are drawn as rectangles. Fragmented sites are labeled by vertical red dashed lines. Gene distribution represented by burgundy rectangles is marked with dark blue lines as gene starts. The X axis is the chromosome position. Scenario (a) is that synteny block was identified after gap threshold was tuned lower. (PNG 67 kb)
Additional file 6: Figure S5. Synteny blocks in *C. elegans* vs. *C. briggsae*. Chromosomes are separated into panels labelled with Roman numeral. The Y axis stands for categories of distribution. Synteny blocks defined by five detection programs: DAGchainer (red), i-ADHoRe (yellow), MCScanX (green), SynChro (light blue), and Satsuma (blue) are drawn as rectangles. The bottom four categories are orthologs between the two species assigned by Opscan (OP; burgundy) and OrthoFinder (OF; purple), and we further categorized orthologs into 1 to 1 orthology (1-1) or many to many orthology (N-N). The X axis is the chromosome position. (PNG 404 kb)
Additional file 7: Table S2. Gene ontology (GO) enrichment analysis of *C. briggsae* genes in synteny break between *C. elegans* and 100kb fragmented *C. briggsae* assemblies. Significant GO terms that appeared in the top 10 ranks of enrichment test either in the original comparison or after assemblies were fragmented, are displayed. The original rank, median rank and number of occurrences that reached top 10 in 100 replications are shown for each GO term. GO terms not belonging to original assembly but reached top 10 after fragmentation are shaded in red. GO:0043066, which was in the original top 10 rank but failed to reach top 10 in all of 100 replications, is shaded in deep red. GO terms belonging to original assembly and remained top 10 after fragmentation are shaded in green. All GO categories were significant after Fisher exact test and have adjusted p-value < 0.01. (DOCX 1 kb)
Additional file 8: Table S3. Assembly statistics among *Caenorhabditis* species and Strongyloides species including ALLMAPS results. (DOCX 1 kb)
