# Supplementary figures and images for: Inferring synteny between genome assemblies: a systematic evaluation
Source: BMC Bioinformatics. 2018 Jan 30;19:26. doi: 10.1186/s12859-018-2026-4 (PMC5791376; doi:10.1186/s12859-018-2026-4)

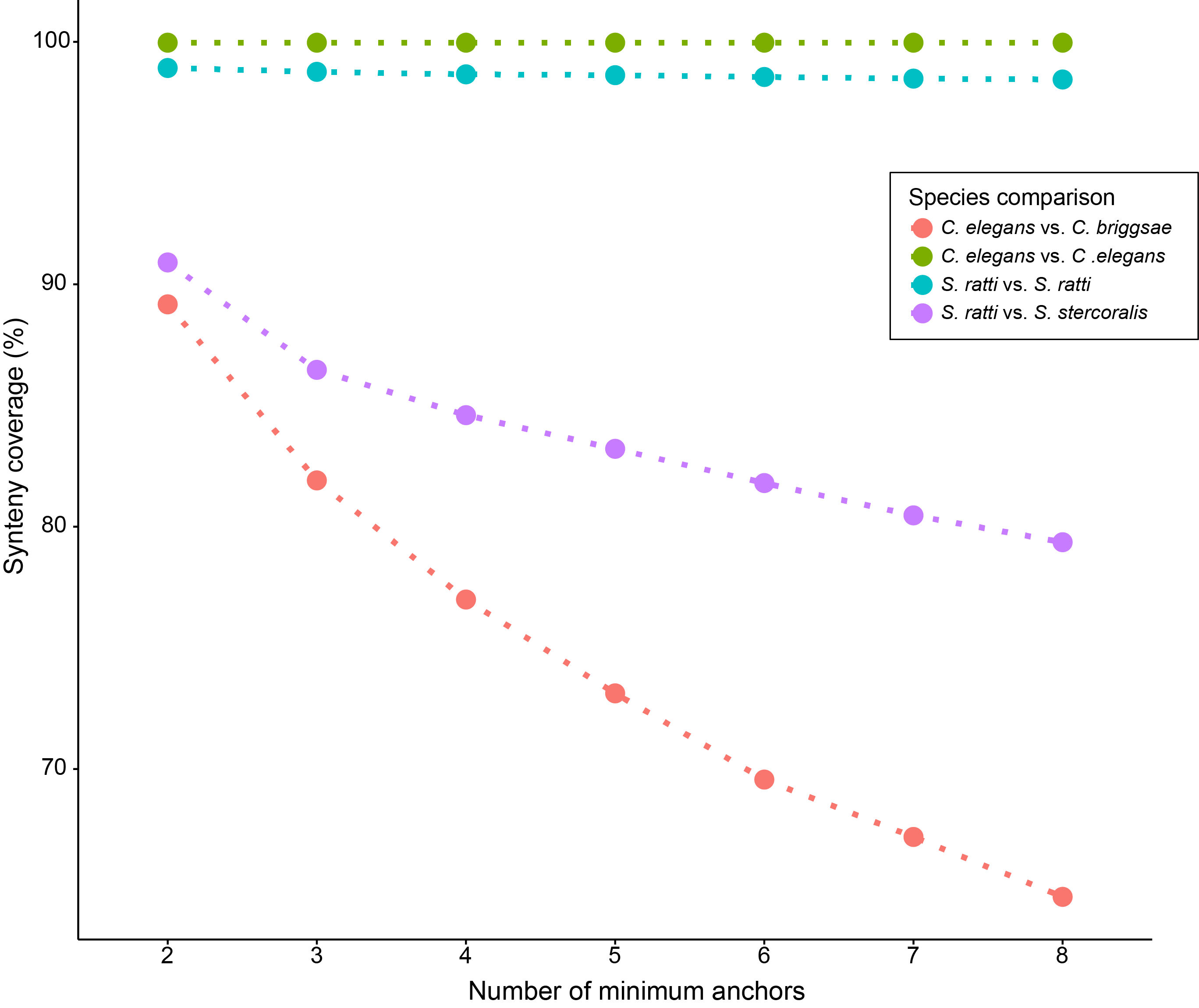

Supplement: Additional file 1: Figure S1. — Synteny coverage for different numbers of minimum anchors using DAGchainer. The Y axis shows synteny coverage (%). The X axis is the number of minimum anchors needed to identify a synteny block from 2 to 8. The 4 colorsare 4 combinations of synteny detection among species: C. elegans vs. C. elegans (CEvsCE, green), C. elegans vs. C. briggsae (CEvsCBG, orange), S. ratti vs. S. ratti (SRvsSR, blue) and S. ratti vs. S. stercoralis (SRvsSS, purple). (PNG 112 kb) [file 12859_2018_2026_MOESM1_ESM.png]

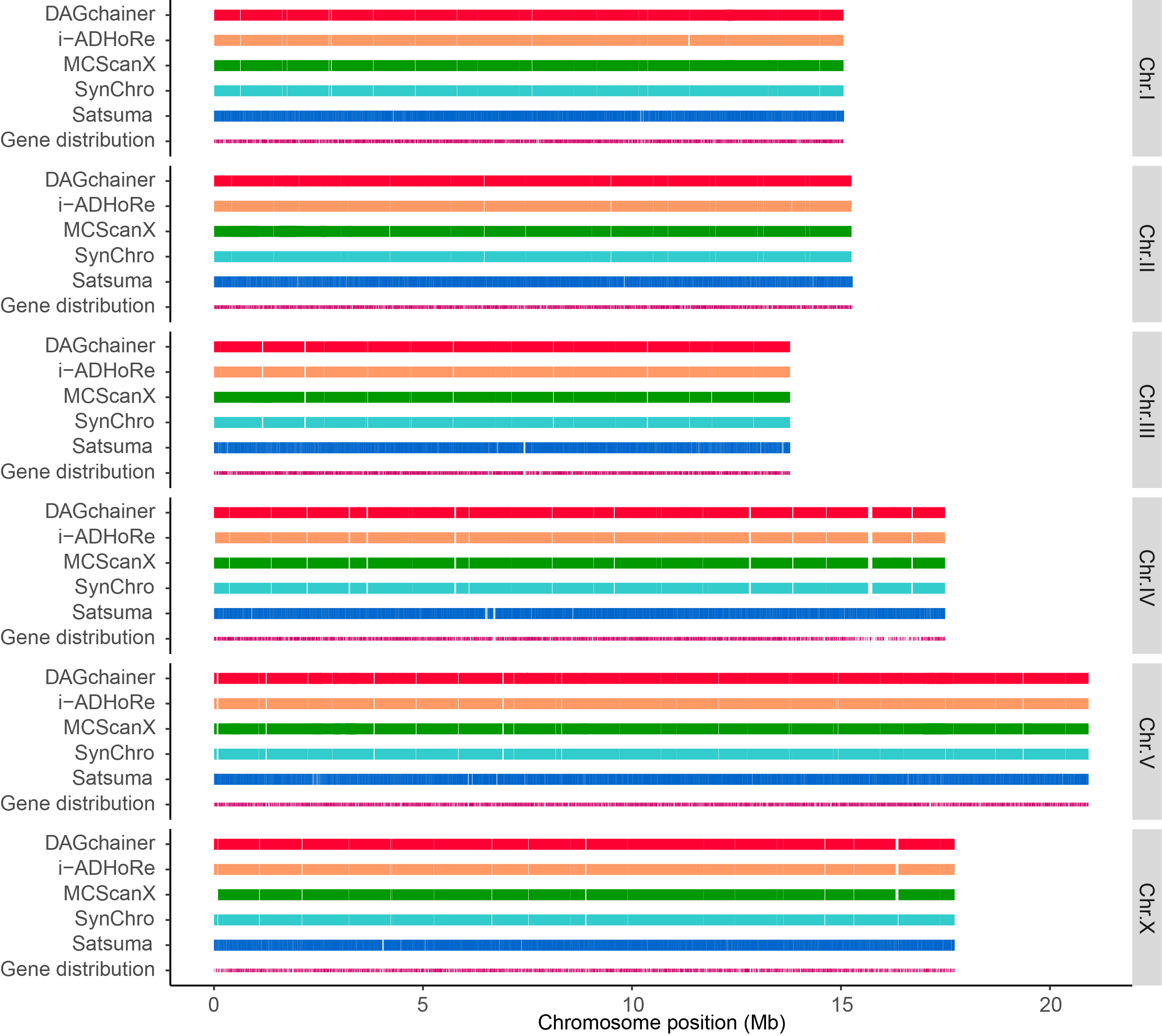

Supplement: Additional file 3: Figure S2. — Synteny blocks in C. elegans vs. 1Mb fragmented C. elegans. Chromosomes are separated into panels labelled with Roman numerals. The Y axis stands for categories of distribution. Synteny blocks defined by five detection programs: DAGchainer (red), i-ADHoRe (yellow), MCScanX (green), SynChro (light blue), and Satsuma (blue) are drawn as rectangles. Gene distribution is represented by the bottom smaller rectangles in burgundy. The X axis is the chromosome position. (PNG 286 kb) [file 12859_2018_2026_MOESM3_ESM.png]

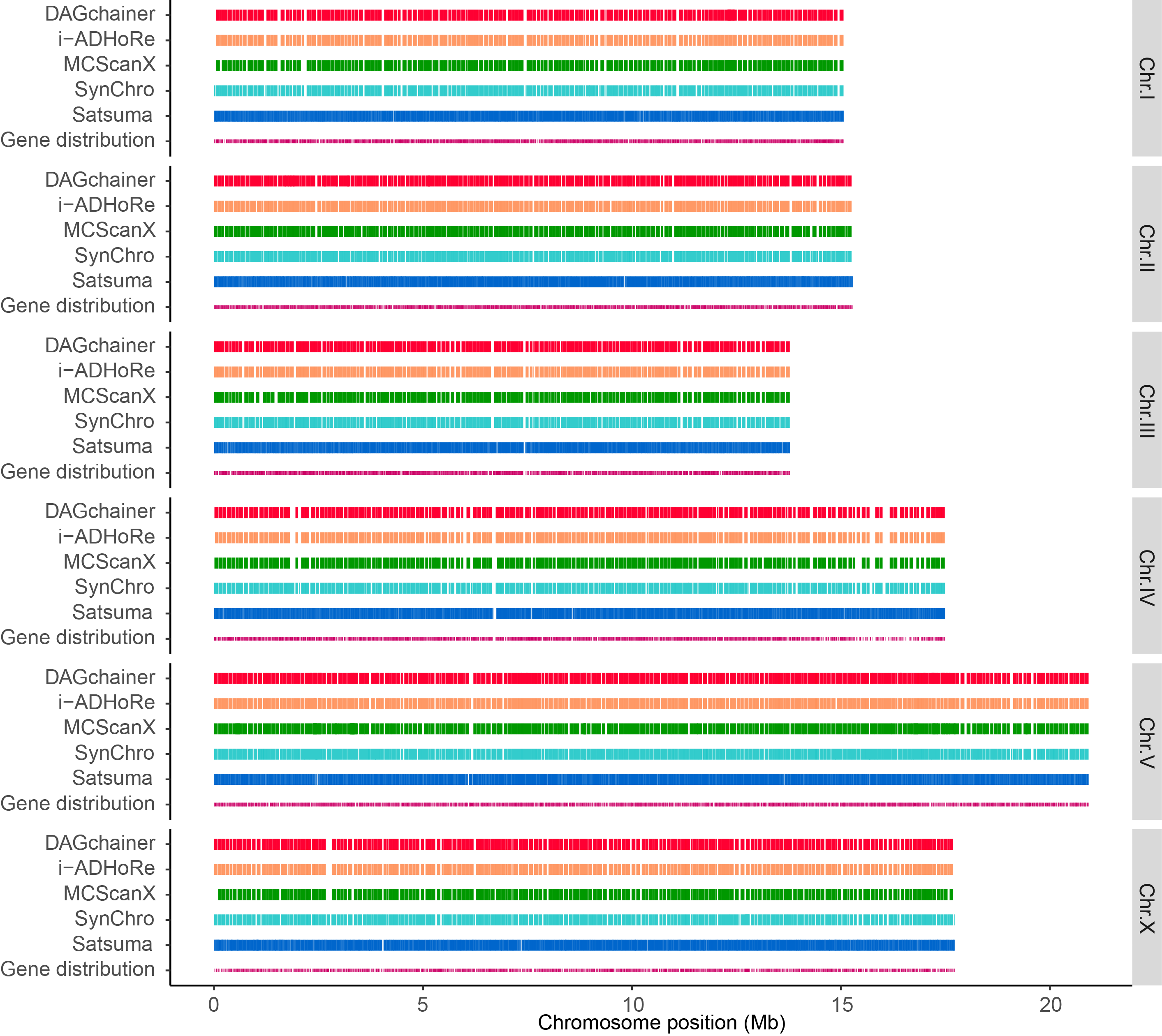

Supplement: Additional file 4: Figure S3. — Synteny blocks in C. elegans vs. 100kb fragmented C. elegans. Chromosomes are separated into panels labelled with Roman numerals. The Y axis stands for categories of distribution. Synteny blocks defined by five detection programs: DAGchainer (red), i-ADHoRe (yellow), MCScanX (green), SynChro (light blue), and Satsuma (blue) are drawn as rectangles. Gene distribution is represented by the bottom smaller rectangles in burgundy. The X axis is the chromosome position. (PNG 322 kb) [file 12859_2018_2026_MOESM4_ESM.png]

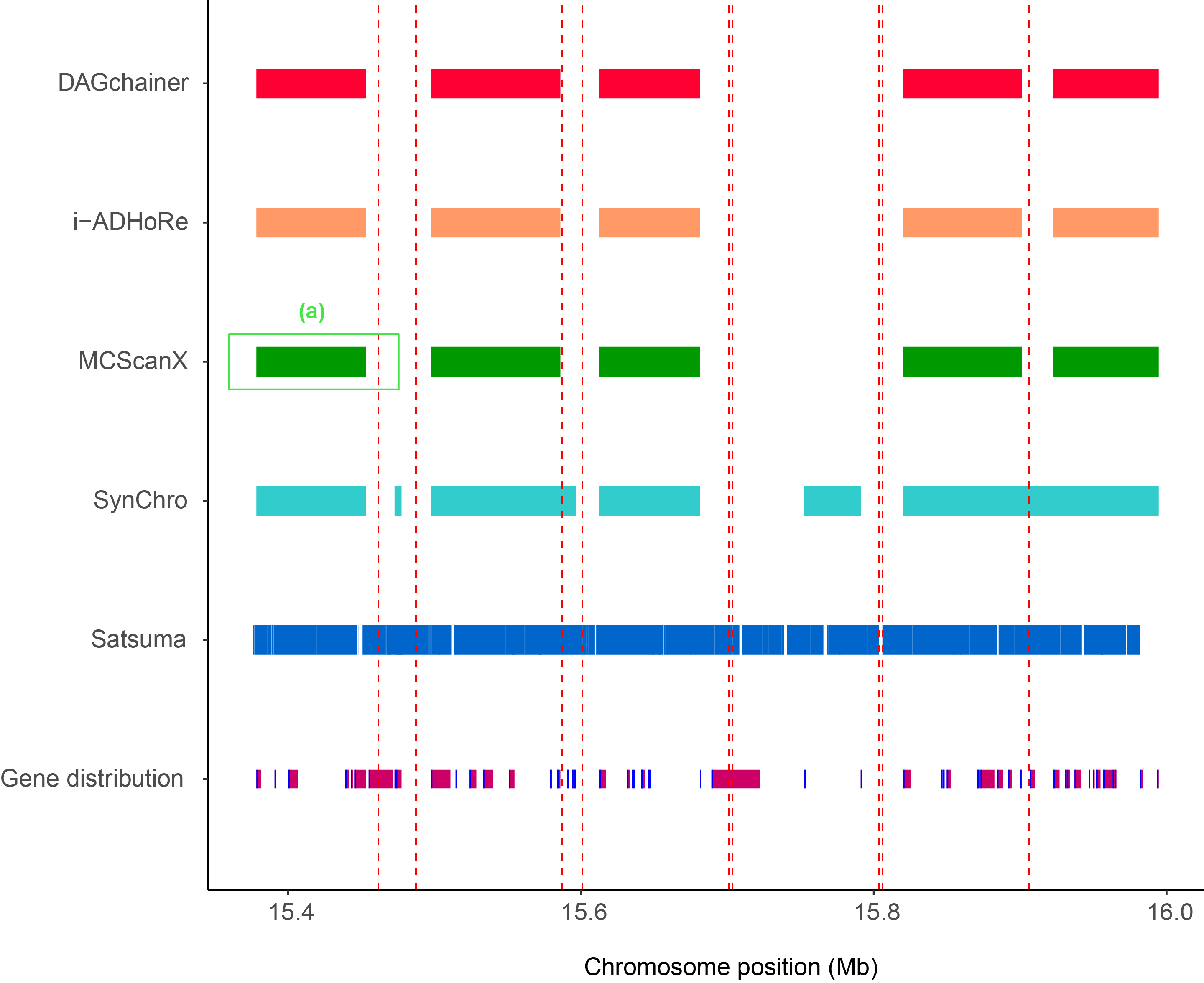

Supplement: Additional file 5: Figure S4. — A zoomed-in 600kb region of synteny identified with lower gap threshold in MCScanX between the reference C. elegans genome and a 100kb fragmented assembly. The Y axis stands for categories of distribution. Synteny blocks in fragmented assembly defined by five detection programs: DAGchainer (red), i-ADHoRe (yellow), MCScanX (green), SynChro (light blue), and Satsuma (blue) are drawn as rectangles. Fragmented sites are labeled by vertical red dashed lines. Gene distribution represented by burgundy rectangles is marked with dark blue lines as gene starts. The X axis is the chromosome position. Scenario (a) is that synteny block was identified after gap threshold was tuned lower. (PNG 67 kb) [file 12859_2018_2026_MOESM5_ESM.png]

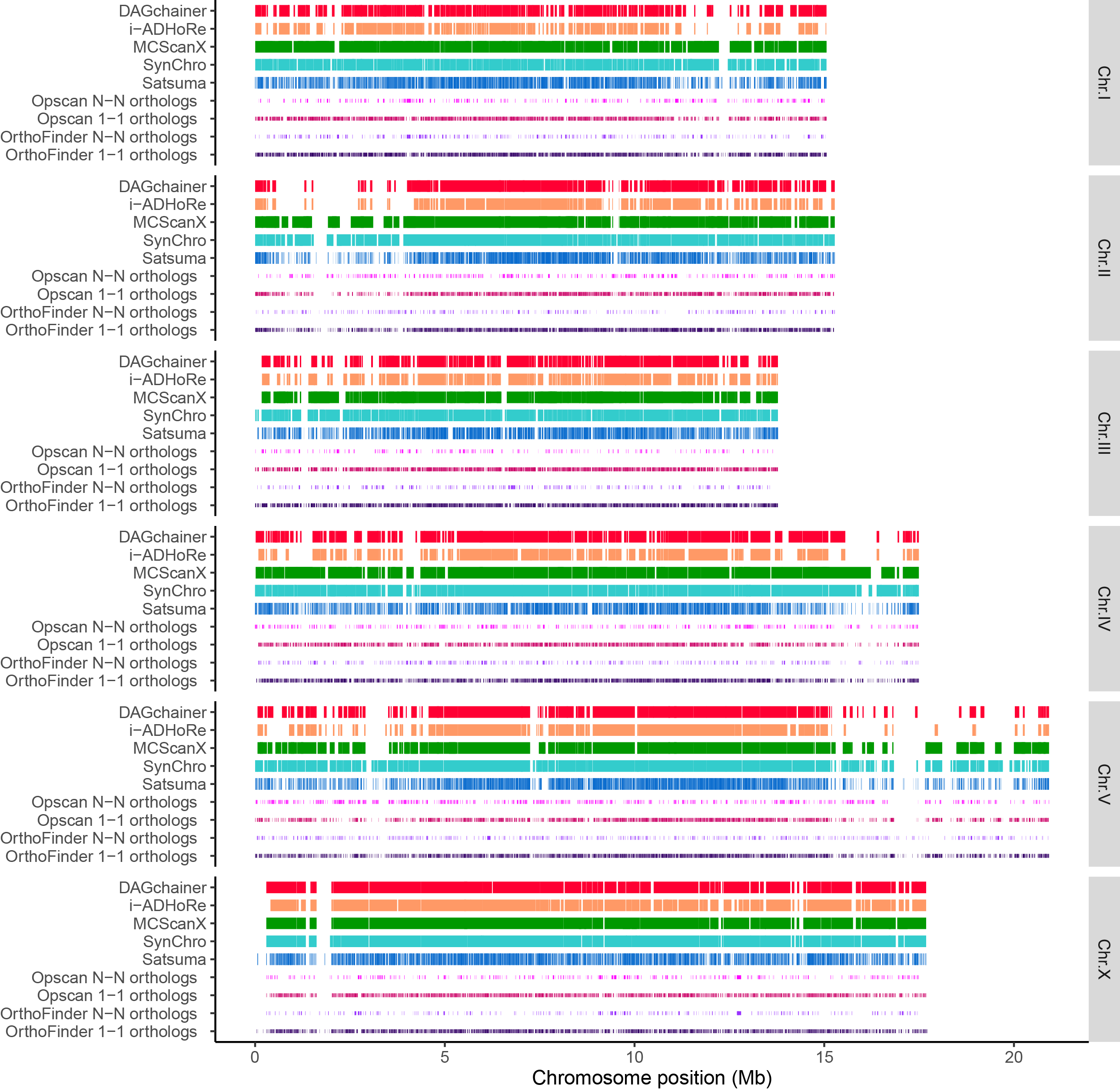

Supplement: Additional file 6: Figure S5. — Synteny blocks in C. elegans vs. C. briggsae. Chromosomes are separated into panels labelled with Roman numeral. The Y axis stands for categories of distribution. Synteny blocks defined by five detection programs: DAGchainer (red), i-ADHoRe (yellow), MCScanX (green), SynChro (light blue), and Satsuma (blue) are drawn as rectangles. The bottom four categories are orthologs between the two species assigned by Opscan (OP; burgundy) and OrthoFinder (OF; purple), and we further categorized orthologs into 1 to 1 orthology (1-1) or many to many orthology (N-N). The X axis is the chromosome position. (PNG 404 kb) [file 12859_2018_2026_MOESM6_ESM.png]
